# Nitric Oxide Stroke Volume Index as a New Hemodynamic Prognostic Parameter for Patients with Pulmonary Arterial Hypertension

**DOI:** 10.3390/jcm9092939

**Published:** 2020-09-11

**Authors:** Karolina Barańska-Pawełczak, Celina Wojciechowska, Mariusz Opara, Wojciech Jacheć

**Affiliations:** 1 Department of Cardiology, Szpital Specjalistyczny, 41-800 Zabrze, Poland; mariusz.opara867@gmail.com; 2 Second Department of Cardiology, School of Medicine with the Division of Dentistry in Zabrze, Medical University of Silesia, 40-055 Katowice, Poland; wojciechowskac@wp.pl (C.W.); wjachec@interia.pl (W.J.)

**Keywords:** arterial pulmonary hypertension, nitric oxide reversibility test, stroke volume index

## Abstract

The aim of the study was to determine the prognostic value of hemodynamic parameters measured during initial diagnostic right heart catheterization (RHC) in standard conditions and using a nitric oxide reversibility test. A retrospective observational study of 62 patients with pulmonary arterial hypertension (PAH) was performed. Clinical, biochemical, echocardiographic, and hemodynamic data obtained at the time of the PAH diagnosis were precisely analyzed. Patients were followed for five years. Death or lung transplantation was considered as a primary endpoint. The mean follow-up period was 1090 ± 703 days and the median age was 46.84 years. In the studied group, 25 patients survived, 36 patients died, and one underwent a lung transplantation. From all the examined parameters, only stroke volume index during reversibility test with iNO (SVI_(NO test)_) (HR = 0.910; 95% confidence interval 0.878–0.944; *p* < 0.001) and initial arterial oxygen saturation (SaO_2_) (HR = 0.910; 95% confidence interval 0.843–0.982; *p* = 0.015) have been established as independent predictors of death or lung transplantation in the five-year follow–up. An SVI_(NO test)_ value above 39.86 mL/m^2^ was associated with 100% five-year survival rate (AUC = 0.956; 95% confidence interval 0.899–1.000; *p* < 0.001; specificity/sensitivity: 100/84%). The results of the analysis suggest that the SVI_(NO test)_ measured during the initial diagnostic RHC could be a very valuable prognostic factor in the PAH patients.

## 1. Introduction

Pulmonary arterial hypertension (PAH) is a progressive, incurable disease which, according to current European Society of Cardiology (ESC) guidelines, constitutes the first group of pulmonary hypertension (PH) clinical classification [1]. It is a heterogeneous group with various of etiologies, however changes in the pulmonary circulation system remain common. Pulmonary arterial intima and media exhibit fractional thicknesses in the PAH patients, which leads to the reduction of pulmonary arteries luminal area and correlates with both the pulmonary vascular resistance (PVR) (*p* < 0.05) and mean pulmonary artery pressure (PAPm) (*p* < 0.05) [2]. Any significant remodeling in pulmonary veins was not found in the PAH patients [3].

Ernst Von Romberg is the author of the first mentioned changes in pulmonary circulation characterized as the “pulmonary vascular sclerosis” which he described in 1891. The following years were a period of arduous search for the etiology and pathophysiology of what researchers at that time saw as a clinical manifestation of PH but it wasn’t until 1973 that the World Health Organization (WHO) set up a group of experts in order to systematize the existing knowledge about PH and standardize clinical and pathological nomenclature [4]. Only 2.8 years was the median survival for patients with idiopathic PAH (IPAH) since diagnosis, however the invention of drugs affecting the following pathways, prostacyclin, endothelin, or nitric oxide, significantly improved the comfort of life and extended survival time [5]. Recent reports from Japan determine a transplant-free survival in five-year observation period at 74% [6] but still, despite significant improvement in survival over the decades, PAH remains a big therapeutic challenge.

Intensive research is underway on new drugs and combination therapies, but equally great attention is being paid to the regular assessment of patients with PAH. Right heart catheterization (RHC) variables currently recommended by ESC to evaluate one-year mortality are: right atrial pressure (RAP), cardiac index (CI) and mixed venous oxygen saturation (SvO2). Value of the RAP < 8 mmHg, CI ≥ 2.5 L/min/m^2^ and SvO2 > 65% enable to classify patients with PAH to the group with low death risk (<5%) whereas the RAP > 14 mmHg, CI < 2.0 L/min/m^2^ and SvO2 < 60% has characterized the group with high death risk (>10%) [1]. There are many other RHC measurements and parameters calculated from them, with correlation to increase mortality, which are not included in current recommendations [7]. Attempts were also made to determine prognostic factors not only at the time of diagnosis, but after the initial therapy [8,9,10]. Despite the huge amount of available data, the haemodynamic parameters recommended for prognosis assessment are still limited.

PAH patients require special care from the moment the diagnosis is made, with a strong emphasis on capturing the moment of deterioration of cardiovascular function. Therefore, the main aim of this study was to evaluate hemodynamic parameters measured in the initial RHC, both in a normal environment and with the reversibility test using inhaled nitric oxide (iNO) as prognostic factors.

## 2. Methods

### Study Population

A single center retrospective study was conducted in the 2nd Department of Cardiology in Zabrze, Medical University of Silesia in Katowice, Poland in which patients with PAH diagnosed and treated between 1994 and 2013 were enrolled.

The inclusion criteria were confirmed in RHC diagnosis of arterial pulmonary hypertension, performed pulmonary arterial hypertension reversibility test with iNO at the diagnostic stage of RHC, completed by five-year follow-up.

The exclusion criteria comprised patients <18 years of age during diagnostic RHC, uncorrected congenital heart disease, Eisenmenger syndrome and any life-threatening disease other than PAH such as active malignancy of any type or history of a malignancy within previous five years, history of unstable angina, myocardial infarction or stroke within 3 months prior diagnostic RHC, significant renal dysfunction, defined as serum creatinine >250 μmol/L (>2.8 mg/dL), liver disease, defined as any liver function tests >3 times the upper limit of normal. The study protocol was reviewed and approved by the Bioethics Committee of the Medical University of Silesia. The endpoints of the study were death or urgent lung transplantation in the five-year follow-up.

## 3. Clinical Assessments

Clinical assessment included: physical examination, pharmacological treatment, six-minute walk test distance (6MWT), and the WHO functional class (WHO-FC).

### 3.1. Echocardiography

Echocardiography images were acquired in standard views recommended by the American Society of Echocardiography and the European Association of Cardiovascular Imaging, and following parameters were measured and calculated: right ventricle end-diastolic diameter (RV-d), right atrium area (RAA), tricuspid annular plane systolic excursion (TAPSE), maximum velocity of tricuspid regurgitation (TR V max.), right ventricle systolic pressure (RVSP), pulmonary flow acceleration time (AcT), vena cava inferior diameter (VCI), and its collapse >50% during inspiration. The presence of fluid in pericardium was also determined.

### 3.2. Biochemical Methods

Concentrations of hemoglobin, sodium, uric acid, and creatinine were determined by the routine techniques. N-terminal pro-brain natriuretic peptide (NT-proBNP) concentration was measured by the chemiluminescence method on Roche Cobas 6000e501 (Roche Diagnostics GmbH, Mannheim, Germany).

### 3.3. Right Heart Catheterization

All patients underwent RHC, which was performed via right jugular access under local anesthesia and in a supine position at rest. A flow-directed Swan–Ganz catheter (Edwards Lifesciences, Irvine, CA, USA) was advanced to the right heart and pulmonary artery under fluoroscopy. After 10 min of stabilization of circulation following parameters were measured: RAP, pulmonary artery pressures systolic (PAPs) and diastolic (PAPd), pulmonary artery wedge pressure (PAWP). All the measurements were performed during end expiration. CI was measured by thermodilution, using a rapid bolus injection of 10 cc of cold saline. Blood pressure was measured non-invasively. PAWP and RAP was measured two times, before and after CI measurements, systolic blood pressure (SBP) and diastolic blood pressure (DBP) was measured three times: before, during third and after fifth CI measurements. Two samples of mixed venous blood for gas analysis were collected. The degree of oxygenation of arterial blood was evaluated using a pulse oximeter.

Mean values of RAP, PAPs, PAPd, PAWP, and heart rate (HR) were used for final evaluation. Acquired data enabled calculation of PAPm and mean blood pressure (MBP), transpulmonary gradient (TPG), pulmonary vascular resistance index (PVRI) and SVI. Body surface area was calculated by angiograph software including patient’s height and weight.

Blood pressure parameters were expressed in millimeters of mercury (mmHg), CI as liters per minute (L/min/m^2^), and heart rate as a number of heart beats per minute (bpm). PVRI was expressed in WU × m^2^ and stroke volume index as milliliters per square meter (mL/m^2^).

## 4. Protocol of Vasoreactivity Testing

The hemodynamic measurements were carried out at baseline air conditions and during testing with iNO 60 parts per million (ppm). iNO (Messer Nitric Oxide 800 ppm mol/mol, Messer Group GmbH Germany or iNOmax 400 ppm mol/mol, INO Therapeutics AB, Lidingö, Sweden) was administered through the nasal mask in a mixture with oxygen (10 L/min.) from a source tank equipped with a microflow gauge (Linde France SA, Malmaison, France).Ten minutes of iNO inhalation were performed before consecutive hemodynamic measurements. At each measuring point, five results were acquired and then an arithmetic mean was calculated. A positive acute response was detected if the reduction of the PAPm ≥ 10 mmHg and reached an absolute value of PAPm ≤ 40 mmHg with an increased or unchanged cardiac output (CO).

## 5. Data Analysis

Factors potentially influencing the prognosis of the PAH patients were analyzed in a subgroup of 62 patients selected according to the above-mentioned inclusion and exclusion criteria. The patients were divided into two groups: the first group comprised patients who survived the five-year follow-up (*n* = 25) and the second group, patients who died or had a lung transplant in this period (*n* = 37).

### Statistical Analysis

The distribution of all continuous variables normally distributed was evaluated by the Shapiro–Wilk test. The continuous data not normally distributed are presented as a median with the first and fourth quartiles and were compared with the Mann–Whitney U test. The continuous variables with a normal data distribution of the data are presented as the mean ± standard deviation (SD) and were compared with the *t*-Student test. Data from the pulmonary hypertension reversibility test were compared depending on their distribution with the paired t-Student test or Wilcoxon paired test. Categorical data are presented as absolute numbers and percentages and were compared using Chi-square test with Yates correction.

Cox proportional hazards regression analysis was applied to identify the variables associated with long-term outcome. All demographic, clinical, echocardiographic, and hemodynamic variables were included in a univariate Cox analysis but only complete unrelated variables with a value of *p* ≤ 0.1 at univariate analysis were included to multivariate Cox proportional hazards regression model with stepwise algorithm selection (*p* < 0.2). The results of the Cox analysis were reported as relative risks with corresponding 95% confidence intervals.

The receiver operating characteristic (ROC) curve was used to measure the cut off value of the significant prognostic factors.

Survival analysis of all deaths or lung transplantations, based on Kaplan-Meier curves and log-rank tests were used to assess the event-free survival between groups of patients separated on the basis of the cut-off points determined by the ROC curve analysis.

The results were considered statistically significant if *p* < 0.05. Lack of statistical significance was presented as NS (non-significant). Statistical analysis was performed using STATISTICA 13.1 PL (StatSoft Inc., Tulsa, OK, USA).

## 6. Results

A total of 62 (53 female) patients in mean age 44.55 ± 15.41 met the inclusion criteria. During the five-year follow-up 36 patients died and one patient underwent lung transplantation. Patients who survived the five-year observation period initially were in the lower WHO-FC, passed a significantly longer distance in 6MWT, and were borderline significant more often responders in the reversible pulmonary hypertension test compared to patients who had died. In addition, they had lower serum concentrations of NT-proBNP, uric acid, and creatinine, as well as a higher sodium concentration. In the group of patients, echocardiography showed smaller RV-d, RAA, TR V max., and TAPSE. There were no differences in AcT values. For survivors the VCI collapsed during inspiration in all cases but in the group of death below five years of observation only for 14.8% such dependence was detected. There were no statistically significant differences between the groups in the etiology of pulmonary hypertension and pharmacotherapy (Table 1).

In the whole group of patients during iNO inhalation (60 ppm) statistically significant improvement was observed for all measured parameters except for PAWP and CI (Table 2).The survivors had both in the initial and iNO measurements a significantly higher SVI, CI, as well as lower HR compared to the group of patients who have died during follow-up. Additionally, survivors had, in initial measurements, higher arterial oxygen saturation (SaO_2_) and SvO_2_ compared to patients who died during the follow-up (Table 3).

### 6.1. Univariable Cox Regression Analysis

For complete data, *p* ≤ 0.1 is considered significant for univariate Cox analysis. The factors that reached this included WHO FC, response for iNO inhalation, distance in 6-MWT, sodium serum concentration, RVd, RAA, TR Vmax., presence of fluid in pericardium, HR_(baseline)_, CI_(baseline)_, SVI_(baseline)_, SaO_2_, SvO_2_, HR_(NO test)_, CI_(NO test)_, and SVI_(NO test)_. For incomplete data, in analogical analysis, *p* ≤ 0.1 have been reached by NT-proBNP and uric acid serum concentration, AcT, and presence of VCI collapse during inspiration (Table 4, Table 5 and Table 6).

### 6.2. Multivariable Cox Regression Analysis with a Step-Wise Algorithm—Selection of Variables for Analysis and Results

**Figure 1:**
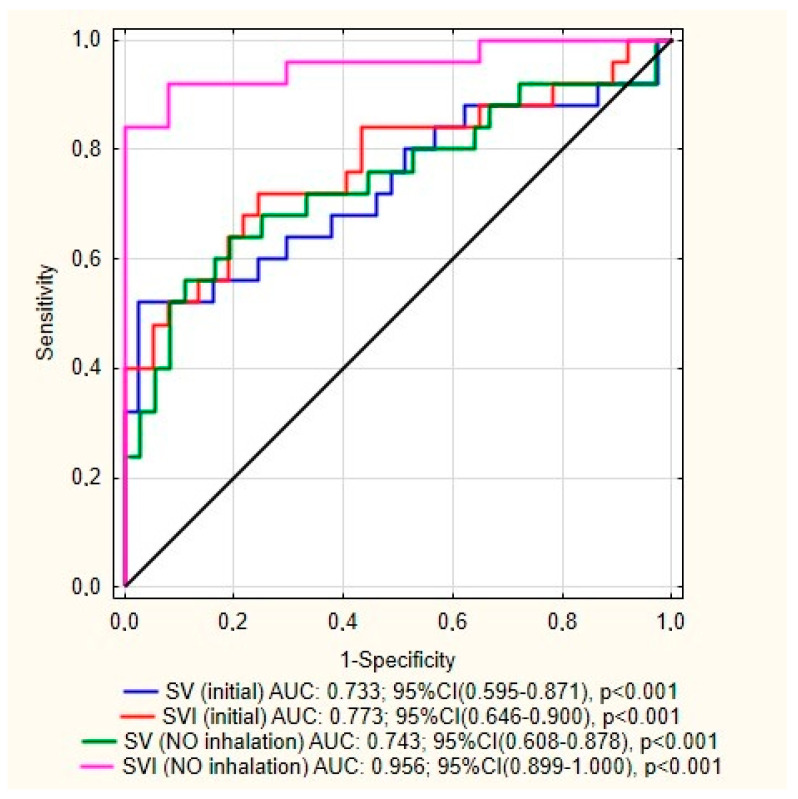
Comparison of ROC curves of dependency between SV along with SVI (obtained during initial measurements and iNO inhalation) and end points occurrence in five-year follow-up.

Bearing in mind that HR, CI, and SVI are strongly related variables and this relationship also exists between SVI_(baseline)_ and SVI_(NO test)_ for multivariate analysis, we decided to include SVI_(NO test)_. In comparison to the SVI_(baseline)_ in the analysis of ROC curves, this parameter has got much higher predictive value. Results of ROC analysis are presented in Figure 1 and Table 7.

The results of multivariable Cox regression analysis of complete data with a step-wise algorithm have shown that only SVI_(NO test)_ and SaO_2_ are independent predictors of worse prognosis. Both higher SVI_(NO test)_ by 1 mL and resting SaO_2_ by one pp were connected with a 9.00% probability reduction of death or lung transplantation in five-year follow-up (Table 8).

Three-factor Cox regression models built on the basis of SVI_(NO test)_ and SaO_2_ did not show the usefulness of uric acid and natrium serum concentration, VCI collapsing during inspiration and AcT as prognostic factors in the studied group of patients. On the other hand, the inclusion of the NT-proBNP serum concentration to the regression model analysis abolished the SaO_2_ prognostic value with the unfavorable, statistically borderline (*p* = 0.063) prognostically higher prognostic value of the NT-proBNP serum concentration, while the SVI_(NO test)_ hazard rate was unchanged (Table 9).

**Figure 2:**
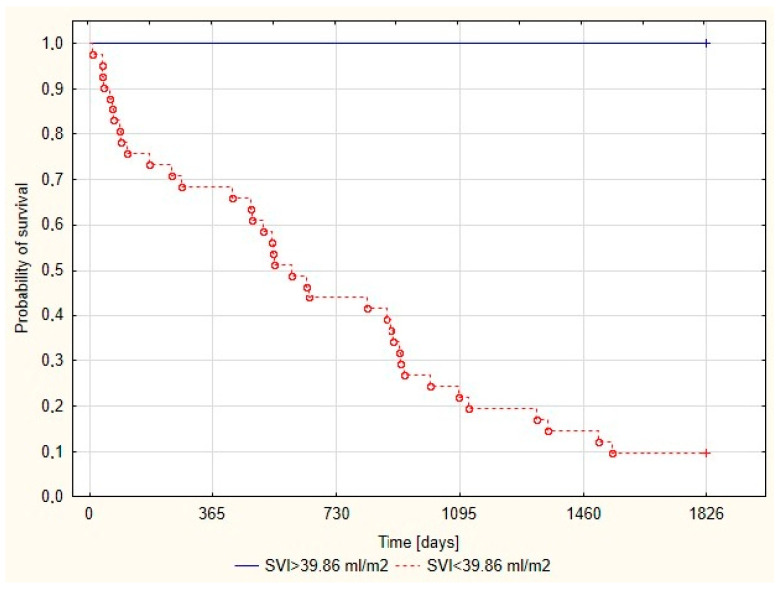
Probability of survival of time free of death or urgent lung transplantation depending on SVI value during iNO inhalation test.

Kaplan-Meier survival curve has been estimated depending on the SVI_(NO test)_ value, with the cut-off point = 39.86 mL/m^2^ (Figure 2). SVI_(NO test)_ above cut-off point is associated with a 100% five-year survival rate.

## 7. Discussion

The univariable analysis confirmed the previously described relationships between worse prognosis and many clinical, laboratory and hemodynamic parameters (Table 4, Table 5 and Table 6). Among hemodynamic measurements recommended in the current guidelines for assessing the risk of one-year mortality for patients with PAH, the predictive value has been confirmed for CI, however such a relationship was not found for RAP. A key finding of this study is that among parameters obtained during the initial RHC only SVI_(NO test)_ and SaO_2_ were recognized as independent predictors of death or lung transplantation in multivariable Cox regression, however SaO_2_ despite its high specificity has had low sensitivity. SV seems to be a new, very valuable prognostic parameter, both for RHC carried out under standard conditions and in the reversibility test with NO. ROC curve analysis results are similar for results of SV obtained during initial measurements and iNO inhalation along with SVI in normal environment, however the attention is drawn to a high predictive properties of the SVI_(NO test)_ value.

SV is a parameter which combines the prognostic value of two parameters: CO (and CI as mentioned above) and HR. Increasing HR is a predictor of worse prognosis for the patients with PAH [11,12,13]. It is proved that the each increase of HR per 10 bpm increases the risk of death, not only at the first electrocardiography made during diagnosis (hazard ratio 1.76: 95% confidence interval 1.42 to 2.18; *p* < 0.001) but also and at the second ECG after the initial treatment (hazard ratio 2.31; 95% confidence interval 1.58 to 3.38; *p* < 0.001) [14].

Patients with PAH have got reduced RV ejection fraction, which decreases even more with disease progression and during exercise. These factors lead to SV reduction. The mechanism adjusted to keep SV stable is a RV enlargement, but the compensation possibilities are limited. This is why increased heart rate through sympathetic hyperactivity [15] is one of the more important mechanisms adjusted to keep CO at a level adequate to the normal demand [16]. Considering the physiology of the pulmonary circulatory system, the above data suggest that the prognostic value of CO and HR cannot be considered separately since the CO may have a normal value at the same time with significantly reduced SV and high HR. It is preferred to use SV indexed to body surface area (*BSA*) as SVI, which allows better comparison of parameters between patients.

Despite the common knowledge about the physiology of pulmonary circulation, data on the usefulness of SVI are limited. One of the first works that confirmed usefulness of SVI was the study of van Wolferen et al. where for patients with pre-capillary PH (PAPm ≥ 25 mmHg and PAWP ≤ 15 mmHg) has been confirmed in magnetic resonance measurements that a baseline SVI > 25 mL/m^2^ (median value) is associated with significantly better survival (log-rank test, *p* < 0.010) [17]. Weatherald et al. has found that SVI under 38 mL/m^2^ (area under the curve 0.68; 95% confidence interval, 0.64–0.72; *p* < 0.01) in the first follow up is a variable associated with increased risk of the death or transplantation and each reduction of SVI per 10 mL/m^2^ increases the risk of adverse event 1.28 times (95% confidence interval, 1.11–1.49; *p* = 0.001).It should be noted that a low SVI still identified patients with poorer prognosis even when they had CI ≥ 2.5 L·min^−1^·m^−2^ [10].

The SVI_(NO test)_ prognostic value has not been considered so far and to understand its predictive value it is necessary to refer to the physiology of vasoreactivity testing. Inhaled iNO through diffusion directly into the smooth muscle of pulmonary vessels can cause a selective local pulmonary vasodilatation without reducing the systemic arterial pressure [18]. The presence of hemoglobin is responsible for iNO inactivation and its local activity [19]. The results obtained during testing with iNO for patients with PAH not only allow to extract a group of patients who will benefit from using calcium channel blockers (CCB) but also provide additional information on prognosis because lack of vascular reactivity is one of the risk factors of death or lung transplantations. It should be emphasized that although SVI already shows prognostic value in standard conditions, SVI_(NO test)_ allows for an additional assessment of potential reserve in improving the function of pulmonary circulation and hence right ventricular functions exist. In regard to the examined population of patients in the group with five-year survival, a higher prevalence of patients with reversible pulmonary hypertension is seen (24% vs. 5.41%). However, being a non-responder does not mean that a five-year observation period will not be achieved, and being a responder is not an absolute indicator of good prognosis. It should be remembered that patients with pulmonary hypertension mainly die from right ventricular failure as a consequence of pulmonary circulation remodeling and SVI is an indicator directly related to the right ventricular function. The SVI value is an objective parameter that allows assessing the severity of heart failure, and the SVI_(NO test)_ below 39.86 mL/m^2^ was established as an independent factor of worse prognosis. All patients with SVI_(NO test)_ above the cut-off point completed a five-year follow-up period. SVI assessed at the time of diagnosis of the pulmonary hypertension allows to isolate at the very beginning a group of patients with particularly poor prognosis. Taking into account the fact that vascular remodeling in PH may be partly reversible through a reduction of PAP, the hemodynamic stress in pulmonary arteries [20] should be considered in terms of how intensive treatment should be administrated at the time of diagnosis in order to slow down the progression as much as possible. Although sequential therapy using one of the following drugs: phosphodiestrase 5 inhibitors (PDE-5i), stimulator of soluble guanylate cyclase (sGC) or endothelin receptor antagonists (ERA), and prostanoids is currently the most commonly used therapeutic strategy [1], the amount of evidence supporting the benefits not only from the initial triple combination therapy [21,22], but also from a rapid achievement of high doses of prostanoids is increasing [23].

When assessing work limitations, the low population size should be mentioned, as well as the fact that, until 2007, treatment options for PAH were very limited in Poland. Available drugs were only PDE-5i (which was not refunded by the government and was often beyond the financial abilities of patients) and CCB. After 2007, the therapeutic options for patients with PAH expanded but still they depended on administrative requirements and not on the patient’s clinical condition.

## 8. Conclusions

Despite a significant improvement in the survival rate of patients with PAH, it remains a disease not only significantly shortening the expected survival time, but also affecting the quality of life. There is no treatment that could completely reverse the pathomorphological changes occurring in the pulmonary circulation and thus cure the disease. Hence, the present therapeutic options are mainly focused on the pharmacological inhibition of major pathways and heart failure treatment. In the current situation, it is particularly important to assess the severity of cardiovascular dysfunction in order to capture the moment when it is necessary to intensify or change the treatment. SVI_(NO test)_ measured during the initial diagnostic RHC seems to be an early parameter with a large prognostic and therapeutic potential, but studies on a large population are essential.

## Figures and Tables

**Table 1:** Basic characteristic of examined clinical, laboratory and echocardiographic data in all group and subgroups separated depending on the prognosis in the five-year follow-up.

	All Group N (%) Mean ± SD Median (IQR) *n* = 62	A 5-Years Survival N (%) Mean ± SD Median (IQR) *n* = 25	B Death or Lung Transplantation Mean ± SD Median (IQR) *n* = 37	A vs. B “U” Mann-Whitney, *t*-Student, Ch^2^ Tests *p*
**General characteristic**				
Female—*n* (%)	53 (85.48)	23 (92.00)	30 (81.08)	*p* = 0.407
age (years) (18.11–74.87) (median (IQR))	46.84 (33.33–58.02)	46.88 (27.00–52.89)	46.80 (33.56–59.28)	*p* = 0.239
Time of follow-up (days) (7–1825)	1089.92 ± 703.00	1825.00	593.24 ± 456.77	*p* = 0.226
WHO-FC I-II—*n* (%)	12 (19.35)	10 (40.00)	2 (5.41)	*p* = 0.002
WHO-FC III-IV—*n* (%)	50 (80.65)	15 (60.00)	35 (94.59)	*p* = 0.002
NO test responder—*n* (%)	8 (12.90)	6 (24.00)	2 (5.41)	*p* = 0.079
6MWT (m)	318.84 ± 136.64	395.06 ± 132.42	265.92 ± 113.81	*p* < 0.001
**PAH etiology**				
Idiopathic—*n* (%)	49 (79.03)	23 (92.00)	26 (70.27)	*p* = 0.081
Heritable—*n* (%)	3 (4.84)	0 (0.00)	3 (8.12)	*p* = 0.392
Connective tissue disease—*n* (%)	7 (11.29)	2 (8.00)	5 (13.51)	*p* = 0.792
Human immunodeficiency virus infection—*n* (%)	1 (1.61)	0 (0.00)	1 (2.70)	*p* = 0.842
Congenital heart disease (corrected)—*n* (%)	2 (3.23)	1 (4.00)	1 (2.70)	*p* = 0.653
**Pharmacotherapy**				
CCB—*n* (%) (monotherapy—*n* (%))	39 (62.90) (22 (35.48))	15 (60.00) (8 (32.00))	24 (64.86) (14 (37.84))	*p* = 0.904
PDE-5i—*n* (%) (monotherapy—*n* (%))	39 (62.90) 19 (30.65)	17 (68.00) 10 (40.00)	22 (59.46) 9 (24.32)	*p* = 0.302
Prostanoids *—*n* (%) (monotherapy—*n* (%))	18 (29.03) (1 (1.61))	5 (20.00) (0 (0.00))	13 (35.14) (1 (2.70))	*p* = 0.316
ERA—*n* (%) (monotherapy—*n* (%))	5 (8.06) (0 (0.00))	4 (16.00) (0 (0.00))	1 (2.70) (0 (0.00))	*p* = 0.206
PDE-5i + prostanoids—*n* (%)	15 (24.19)	3 (12.00)	12 (37.83)	*p* = 0.124
PDE-5i + ERA—*n* (%)	3 (4.84)	2 (8.00)	1 (2.70)	*p* = 0.753
PDE-5i + prostanoids + ERA—*n* (%)	2 (3.23)	2 (8.00)	0 (0.00)	
**Basic biochemistry**				
NT-proBNP ** (pg/mL) (*n* = 38)	1828.0 (2020.0–3270.0)	2020.0 (880.0–2656.0)	2253.0 (1726.0–3645.0)	*p* = 0.001
Uric acid ** (µmol/L) (*n* = 46)	393.00 (303.00–536.00)	307.00 (273.00–360.00)	474.00 (393.00–570.00)	*p* = 0.003
Haemoglobin (g/dL)	13.85 (12.60–14.90)	13.90 (12.90–14.60)	13.80 (12.30–14.90)	*p* = 0.649
Sodium (mmol/L)	139.00 (136.00–142.00)	140.60 (137.00–143.00)	138.00 (136.00–141.00)	*p* = 0.046
Creatinine (µmol/L)	81.65 (66.80–99.00)	66.80 (61.40–79.20)	90.00 (78.00–106.40)	*p* = 0.001
**Echocardiographic data**				
RVd (4 chamber view) (mm)	43.10 ± 10.57	39.29 ± 11.22	45.57 ± 9.48	*p* = 0.022
RAA (cm^2^)	23.15 ± 8.19	18.54 ± 6.86	26.31 ± 7.57	*p* < 0.001
TAPSE ** (mm) (*n* = 36)	17.42 ± 5.90	18.95 ± 5.89	15.71 ± 5.58	*p* = 0.100
TR V max. (m/s)	4.17 ± 2.71	3.79 ± 2.81	4.41 ± 2.48	*p* = 0.006
RVSP (mmHg)	78.44 ± 29.57	64.60 ± 31.77	87.78 ± 24.58	*p* = 0.001
AcT ** (ms) (*n* = 58)	65.34 ± 24.77	77.39 ± 27.73	57.43 ± 19.20	*p* = 0.648
Fluid in pericardium—*n* (%)	18 (29.03)	4 (14.29)	14 (37.84)	*p* = 0.116
VCI ** diameter (mm) (*n* = 53)	19.00 (16.50–22.50)	18.00 (15.00–21.00)	20.00 (17.00–23.00)	*p* = 0.102
VCI **collapse during inspiration—N1/N2 (%)	15/38 (28.30)	11/11 (100.00)	4/27 (14.80)	*p* = 0.004

* prostanoids (iloprost; *n* = 8, treprostinil; *n* = 7, epoprostenol; *n* = 2), ** data incomplete.6MWT—6 min walk test, AcT—pulmonary flow acceleration time, CCB—calcium channel blockers, ERA—endothelin receptor antagonist, IQR—interquartile range, N1—number of events, N2—total number of measurements, NO—nitric oxide, NT-proBNP—N-terminal pro-brain natriuretic peptide, PDE-5i—phosphodiestrase 5 inhibitors, RAA—right atrium area, RVd—right ventricle diameter (4-chamber presentation), RVSP—right ventricle systolic pressure, SD—standard deviation, TAPSE—tricuspid annular plane systolic excursion, TR V max.—maximumvelocity oftricuspid regurgitation, WHO-FC—World Health Organization functional classification, VCI—vena cava inferior diameter.

**Table 2:** Nitric oxide pulmonary reversibility test results in all group.

	Basic Measurements Mean ± SD Median (IQR)	iNO Inhalation (60 ppm) Mean ± SD Median (IQR)	Wilcoxon Test, Paired *t*-Student Test *p*
PAPs (mmHg)	80.10 (64.00–99.50)	65.00 (54.00–83.50)	*p* < 0.001
PAPd (mmHg)	38.10 (31.00–48.00)	33.50 (24.20–39.10)	*p* < 0.001
PAPm (mmHg)	52.57 (41.67–62.00)	44.00 (33.60–53.50)	*p* < 0.001
SBP (mmHg)	122.67 ± 18.53	120.79 ± 17.70	*p* = 0.023
DBP (mmHg)	75.89 ± 12.18	73.57 ± 12.05	*p* = 0.013
MBP (mmHg)	91.48 ± 12.98	89.44 ± 12.54	*p* < 0.007
HR (bpm)	81.67 ± 14.71	76.75 ± 14.20	*p* < 0.001
SVI (mL/m^2^)	27.09 (20.71–34.69)	28.61 (22.62–56.48)	*p* = 0.058
PAWP (mmHg)	10.00 (6.70–11.00)	9.58 (6.08–11.75)	*p* = 0.433
PVRI (Wood units × m^2^)	19.52 (14.73–29.97)	15.76 (10.85–26.59)	*p* < 0.001
TPG (mmHg)	43.75 (34.77–55.33)	31.90 (22.41–39.40)	*p* < 0.001
RAP (mmHg)	8.00 (5.00–12.00)	7.50 (4.00–11.00)	*p* < 0.001
CI (L/min/m^2^)	2.17 (1.70–2.88)	2.24 (1.86–2.88)	*p* = 0.791

bpm—beats per minute, CI—cardiac output index, DBP—diastolic blood pressure, HR—heart rate, iNO—inhaled nitric oxide, IQR—interquartile range, MBP—mean blood pressure, PAPd—diastolic pulmonary artery pressure, PAPm—mean pulmonary artery pressure, PAPs—systolic pulmonary artery pressure, PAWP—pulmonary artery wedge pressure, PVRI—pulmonary vascular resistance index, RAP—right atrium pressure, SBP—systolic blood pressure, SD—standard deviation, SVI—stroke volume index, TPG—transpulmonary pressure gradient.

**Table 3:** Hemodynamic characteristic of subgroups depending on the prognosis in the five-year follow-up.

	Basic Hemodynamic Parameters			iNO Inhalation Hemodynamic Parameters				
	5-Years Survival Mean ± SD Median (IQR) *n* = 25	Death or Lung Transplantation Mean ± SD Median (IQR) *n* = 37	Mann-Whitney U, *t*-Student, Tests *p*	5-Years Survival Mean ± SD *n* = 25	Death or Lung Transplantation Mean ± SD *n* = 37	Mann-Whitney U, *t*-Student, Tests *p*	Wilcoxon Test, Paired t-Student Test *p*	Wilcoxon Test, Paired *t*-Student Test *p*
	A	B	A vs B	C	D	C vs D	A vs C	B vs D
PAPs (mmHg)	85.00 (62.00–99.50)	80.00 (65.80–98.00)	*p* = 0.763	60.00 (41.25–83.00)	68.40 (57.00–90.00)	*p* = 0.146	*p* = 0.001	*p* = 0.001
PAPd (mmHg)	37.60 (26.00–48.00)	38.20 (33.00–43.00)	*p* = 0.651	29.80 (21.00–39.00)	33.80 (26.00–39.20)	*p* = 0.290	*p* = 0.001	*p* = 0.001
PAPm (mmHg)	55.00 (38.00–62.33)	51.87 (44.33–60.00)	*p* = 0.774	41.17 (27.27–52.67)	45.33 (39.07–55.00)	*p* = 0.202	*p* = 0.001	*p* = 0.001
SBP (mmHg)	124.22 ± 16.02	121.62 ± 20.19	*p* = 0.592	120.77 ± 16.45	120.80 ± 18.78	*p* = 0.994	*p* = 0.080	*p* = 0.157
DBP (mmHg)	74.91 ± 14.24	76.55 ± 10.74	*p* = 0.606	72.21 ± 12.61	74.54 ± 11.72	*p* = 0.465	*p* = 0.093	*p* = 0.072
MBP (mmHg)	91.34 ± 13.86	91.56 ± 12.54	*p* = 0.948	88.40 ± 12.57	90.21 ± 12.65	*p* = 0.588	*p* = 0.049	*p* = 0.069
HR (bpm)	76.04 ± 16.07	85.47 ± 12.55	*p* = 0.012	71.66 ± 14.32	80.39 ± 13.13	*p* = 0.017	*p* = 0.035	*p* < 0.001
SVI (mL/m^2^)	34.69 (25.92–46.88)	21.41 (19.52–29.07)	*p* < 0.001	65.62 (49.20–76.40)	24.41 (17.99–28.27)	*p* < 0.001	*p* = 0.001	*p* = 0.308
PAWP (mmHg)	9.00 (5.00–11.00)	10.00 (8.00–11.00)	*p* = 0.312	8.00 (5.00–12.00)	10.00 (7.00–11.50)	*p* = 0.368	*p* = 0.374	*p* = 0.846
PVRI (Wood units × m^2^)	16.42 (10.93–27.24)	24.52 (15.68–29.97)	*p* = 0.164	12.13 (7.10–21.11)	18.12 (12.74–27.28)	*p* = 0.070	*p* = 0.001	*p* = 0.001
TPG (mmHg)	47.33 (31.22–55.33)	41.87 (37.03–52.00)	*p* = 0.949	32.77 (19.67–45.67)	37.13 (30.07–45.00)	*p* = 0.286	*p* = 0.001	*p* = 0.001
RAP (mmHg)	7.00 (3.70–13.00)	8.00 (7.00–10.00)	*p* = 0.518	6.00 (3.00–12.00)	8.00 (6.00–10.00)	*p* = 0.323	*p* = 0.004	*p* = 0.019
CI (L/min/m^2^)	2.69 (2.02–3.13)	1.98 (1.63–2.50)	*p* = 0.007	2.48 (2.21–3.07)	2.14 (1.72–2.44)	*p* = 0.006	*p* = 0.903	*p* = 0.397
SaO_2_ (%)	94.40 (92.00–97.00)	92.00 (89.00–93.40)	*p* = 0.008					
SvO_2_ (%)	69.00 (65.00–75.00)	64.50 (58.00–71.00)	*p* = 0.014					

bpm—beats per minute, CI—cardiac output index, DBP—diastolic blood pressure, HR—heart rate, IQR—interquartile range, MBP—mean blood pressure, PAPd—diastolic pulmonary artery pressure, PAPm—mean pulmonary artery pressure, PAPs—systolic pulmonary artery pressure, PAWP—pulmonary artery wedge pressure, PVRI—pulmonary vascular resistance index, RAP—right atrium pressure, SaO_2_—arterial oxygen saturation, SBP—systolic blood pressure, SD—standard deviation, SVI—stroke volume index, SvO_2_—mixed venous oxygen saturation, TPG—transpulmonary pressure gradient.

**Table 4:** Risk factors of death or lung transplantations in five-year follow-up. Univariable Cox regression analysis of clinical, echocardiographic and laboratory parameters.

	HR	95% Confidence Interval	*p*
Female (yes/no)	0.507	0.222–1.157	0.107
Age (years)	1.012	0.990–1.035	0.292
WHO–FC (by one class)	2.408	1.458–3.978	0.001
NO test responder (yes/no)	0.258	0.062–1.076	0.063
6MWT distance (1 m)	0.995	0.992–0.997	<0.001
PAH Idiopathic (yes /no)	1.049	0.495–2.225	0.900
PAH Connective tissue disease (yes/no)	1.327	0.552–3.188	0.527
CCB (only) (yes/no)	1.139	0.580–2.239	0.705
PDE-5i (only) (yes/no)	0.783	0.406–1.510	0.465
PDE-5i + prostanoids(yes/no)	1.379	0.701–2.715	0.352
PDE-5i + ERA(yes/no)	0.232	0.032–1.692	0.15
NT-proBNP (by 100 pg/mL)	1.012	1.005–1.019	<0.001
Uric acid (by 10 µmol/L)	1.031	1.013–1.048	<0.001
Haemoglobin (by 1 g/dL)	0.921	0.779–1.088	0.331
Sodium (by 1 mmol/L)	0.927	0.852–1.008	0.075
Creatinine (by 1 µmol/L)	1.018	1.006–1.030	0.002
RVd (4 chamber view) (by 1 mm)	1.028	1.001–1.055	0.041
RAA (by 1 cm^2^)	1.07	1.032–1.110	<0.001
TAPSE (by 1 mm)	0.922	0.922–0.922	0.922
TR V max. (by 1 m/s)	1.924	1.274–2.906	0.002
RVSP (by 1 mmHg)	1.017	1.006–1.029	0.002
AcT (by 1 ms)	0.976	0.961–0.992	0.003
Fluid in pericardium (yes/no)	1.986	1.018–3.874	0.044
VCI diameter (1 mm) (*n* = 53)	0.994	0.964–1.026	0.729
VCI collapse during inspiration (yes/no)	0.258	0.090–0.740	0.012

6MWT—6 min walk test, AcT—pulmonary flow acceleration time, CCB—calcium channel blockers, ERA—endothelin receptor antagonist, HR—hazard ratio, NT-proBNP—N-terminal pro-brain natriuretic peptide, PDE-5i—phosphodiestrase-5 inhibitors, RAA—right atrium area, RVd—right ventricle diameter (4-chamber presentation), RVSP—right ventricle systolic pressure, TAPSE—tricuspid annular plane systolic excursion, TR V max.—*maximum* velocity of *tricuspid regurgitation*, VCI—vena cava inferior diameter, WHO-FC—World Health Organization functional class.

**Table 5:** Risk factors of death or lung transplantations in five-year follow-up. Univariable Cox regression analysis of hemodynamic initial parameters.

	HR	95% Confidence Interval	*p*
PAPs (mmHg)	1.001	0.989–1.013	0.877
PAPd (mmHg)	1.007	0.986–1.028	0.523
PAPm (mmHg)	1.004	0.986–1.021	0.681
SBP (mmHg)	0.991	0.972–1.009	0.318
DBP (mmHg)	1.004	0.979–1.029	0.778
MBP (mmHg)	0.997	0.972–1.021	0.787
HR (bpm)	1.030	1.009–1.052	0.006
SVI (mL/m^2^)	0.939	0.907–0.971	<0.001
PAWP (mmHg)	1.058	0.970–1.155	0.201
PVRI (Wood units × m^2^)	1.017	0.990–1.044	0.220
TPG (mmHg)	1.001	0.983–1.020	0.893
RAP (mmHg)	1.003	0.949–1.060	0.910
CI (L/min/m^2^)	0.500	0.300–0.834	0.008
SaO_2_ (%)	0.874	0.818–0.934	<0.001
SvO_2_ (%)	0.970	0.945–0.996	0.024

bpm—beats per minute, CI—cardiac output index, DBP—diastolic blood pressure, HR—heart rate, MBP—mean blood pressure, PAPd—diastolic pulmonary artery pressure, PAPm—mean pulmonary artery pressure, PAPs—systolic pulmonary artery pressure, PAWP—pulmonary artery wedge pressure, PVRI—pulmonary vascular resistance index, RAP—right atrium pressure, SaO_2_—arterial oxygen saturation, SBP—systolic blood pressure, SVI—stroke volume index, SvO_2_—mixed venous oxygen saturation, TPG—transpulmonary pressure gradient.

**Table 6:** Risk factors of death or lung transplantations in five-year follow-up. Univariable Cox regression analysis of hemodynamic parameters measured during iNO inhalation.

	HR	95% Confidence Interval	*p*
PAPs (mmHg)	1.005	0.995–1.016	0.349
PAPd (mmHg)	1.010	0.990–1.031	0.340
PAPm (mmHg)	1.008	0.992–1.024	0.334
SBP (mmHg)	0.997	0.977–1.017	0.740
DBP (mmHg)	1.006	0.980–1.033	0.643
MBP (mmHg)	1.003	0.976–1.030	0.849
HR (bpm)	1.033	1.009–1.057	0.006
SVI (mL/m^2^)	0.913	0.883–0.945	<0.001
PAWP (mmHg)	1.065	0.985–1.151	0.113
PVRI (Wood units × m^2^)	1.023	0.995–1.052	0.114
TPG (mmHg)	1.010	0.982–1.039	0.496
RAP (mmHg)	1.009	0.953–1.069	0.750
CI (L/min/m^2^)	0.467	0.266–0.818	0.008

bpm—beats per minute, CI—cardiac output index, DBP—diastolic blood pressure, HR—heart rate, MBP—mean blood pressure, PAPd—diastolic pulmonary artery pressure, PAPm—mean pulmonary artery pressure, PAPs—systolic pulmonary artery pressure, PAWP—pulmonary artery wedge pressure, PVRI—pulmonary vascular resistance index, RAP—right atrium pressure, SBP—systolic blood pressure, SVI—stroke volume index, TPG—transpulmonary pressure gradient.

**Table 7:** Receiver operating characteristic curve analysis results.

	AUC	95% Confidence Interval	*p*	Cut Off Value	Specificity/Sensitivity (%)
HR_(baseline)_	0.682	0.540–0.823	0.0118	79.6bpm	72.97/64.00
CI_(baseline)_	0.704	0.568–0.839	0.0032	2.471 L/min/m^2^	72.97/64.00
SVI_(baseline)_	0.773	0.646–0.900	<0.0001	29.142 mL/m^2^	74.48/72.44
SaO_2_	0.699	0.568–0.831	0.0030	95.20%	85.78/48.00
RAA	0.784	0.661–0.907	<0.0001	17.00 cm^2^	94.29/58.33
HR_(NO test)_	0.674	0.530–0.817	0.0178	76.75 bpm	68.57/72.00
CI_(NO test)_	0.71	0.576–0.844	0.0021	2.372 L/min/m^2^	75.00/64.00
SVI_(NO test)_	0.956	0.899–1.000	<0.0001	39.86 mL/m^2^	100.0/84.00

AUC—area under the curve, bpm—beats per minute, CI—cardiac output index, HR—heart rate, NO—nitric oxide, RAA—right atrium area, SaO_2_—arterial oxygen saturation, SVI—stroke volume index.

**Table 8:** Multivariable Cox regression analysis with step wise algorithm (*p* < 0.2) results.

	HR	95% Confidence Interval	*p*
SVI_(NO test)_ (mL/m^2^)	0.910	0.878–0.944	<0.001
SaO_2_ (1%)	0.910	0.843–0.982	0.015

NO—nitric oxide, SaO_2_—arterial oxygen saturation, SVI—stroke volume index.

**Table 9:** Three-variable Cox regression analysis of incomplete data (NT-proBNP, uric acid, sodium, AcT, VCI collapse during inspiration).

	HR	95% Confidence Interval	*p*
SVI_(NO test)_ (mL/m^2^)	0.917	0.876–0.960	<0.001
SaO_2_ (1%)	0.909	0.795–1.040	0.166
NT-proBNP (pg/mL) (*n* = 38)	1.008	1.000–1.016	0.063
SVI_(NO test)_ (mL/m^2^)	0.903	0.864–0.945	<0.001
SaO_2_ (1%)	0.896	0.810–0.990	0.031
Uric acid (µmol/L) (*n* = 46)	1.013	0.992–1.035	0.227
SVI_(NO test)_ (mL/m^2^)	0.911	0.878–0.946	<0.001
SaO_2_ (1 pp)	0.911	0.843–0.984	0.0175
Sodium (mmol/L) (*n* = 62)	0.983	0.906–1.066	0.679
SVI_(NO test)_ (mL/m^2^)	0.910	0.877–0.945	<0.001
SaO_2_ (1%)	0.903	0.836–0.976	0.010
AcT (ms) (*n* = 58)	0.991	0.974–1.008	0.275
SVI_(NO test)_ (mL/m^2^) (*n* = 53)	0.901	0.862–0.943	<0.001
SaO_2_ (1%)	0.904	0.831–0.984	0.019
VCI collapse during inspiration (*n* = 53)	2.279	0.660–7.869	0.193

AcT—pulmonary flow acceleration time, *n*—number of events, NO—nitric oxide, NT-proBNP—N-terminal pro-brain natriuretic peptide, SaO_2_—arterial oxygen saturation, SVI—stroke volume index, VCI—vena cava inferior diameter.
